# Munchausen by proxy syndrome mimicking systemic autoinflammatory disease: case report and review of the literature

**DOI:** 10.1186/s12969-017-0152-6

**Published:** 2017-04-05

**Authors:** Helmut Wittkowski, Claas Hinze, Sigrid Häfner-Harms, Vinzenz Oji, Katja Masjosthusmann, Martina Monninger, Ulrike Grenzebach, Dirk Foell

**Affiliations:** 1 Department of Paediatric Rheumatology and Immunology, University Children’s Hospital Muenster, Albert-Schweitzer-Campus 1, Bld. W30, D-48149 Muenster, Germany; 2 Department of General Pediatrics, University Children’s Hospital Muenster, Muenster, Germany; 3 Department of Dermatology, University Hospital Muenster, Muenster, Germany; 4 Department of Ophthalmology, University Hospital Muenster, Muenster, Germany

**Keywords:** Autoinflammation, Systemic autoinflammatory disease (SAID), Münchhausen by proxy syndrome (MBPS), Differential diagnosis

## Abstract

**Background:**

Systemic autoinflammatory diseases (SAIDs) represent a growing number of monogenic, polygenic or multifactorial disorders that are often difficult to diagnose.

**Case presentation:**

Here we report a patient who was initially erroneously diagnosed and treated for SAID. Symptoms consisted of recurrent fever, erythematous and/or blistering skin lesions, angioedema, susceptibility to bleeding, external ear infections and reversible anisocoria in the absence of laboratory evidence of systemic inflammation. After two and a half years of extensive diagnostic work-up and multiple empirical therapies, a final diagnosis of Munchausen by proxy syndrome (MBPS) was established.

**Conclusions:**

The diagnosis of SAID needs to be carefully reassessed if measurable systemic inflammation is missing, and MBPS should be included in the differential diagnosis.

## Background

Systemic autoinflammatory diseases (SAIDs) comprise of genetically defined conditions as well as disorders of unknown pathogenesis. Examples of monogenic SAIDs are Familial Mediterranean fever (FMF), cryopyrin associated periodic syndromes (CAPS), mevalonate kinase (MVK) deficiency and tumor necrosis factor receptor associated periodic syndrome (TRAPS). In cases where a specific diagnosis cannot be achieved, an undifferentiated SAID is assumed. In contrast to autoimmune diseases, SAIDs are driven by uncontrolled activation of the innate immune system [1, 2]. Typical clinical features include flares of recurrent inflammation, fever, skin rash, arthritis, serositis and organ involvement (e.g. skin, eyes, kidney). The clinical presentation of these disorders overlaps to a certain degree, complicating and delaying their diagnosis. Moreover, co-factors responsible for genotype-phenotype variations are hard to identify, which can delay diagnosis further [3].

Munchausen by proxy syndrome (MBPS) in children, first described in 1977 by Meadow, refers to a fabricated or induced illness [4, 5]. Alternative terms for MBPS include factitious disorder by proxy or medical child abuse. Regardless of the nomenclature, MBPS is defined as harm carried out by a caregiver, usually the mother, where a medical condition is exaggerated, fabricated or induced [6]. Perpetrators often have medical knowledge or ties to the medical sector, may suffer from somatoform or factitious disease or personality disorders, or intend to gain social attention or raise money for personal use [5]. MBPS can be seen as a variant of battered child syndrome (BCS). While BCS is an important cause of overall paediatric morbidity and mortality with more than 650 thousand victims per year in the US, MBPS is actually a rare entity with an annual incidence of approximately 0.5/100.000 in children younger than 16 years, though it is likely to be underreported [5, 7]. The spectrum of systemic involvement with which MBPS may present is very broad and includes dermatologic, endocrine, gastrointestinal, hematologic, neurologic, metabolic, infectious or rheumatologic symptoms and fever.

## Case presentation

**Fig. 1:**
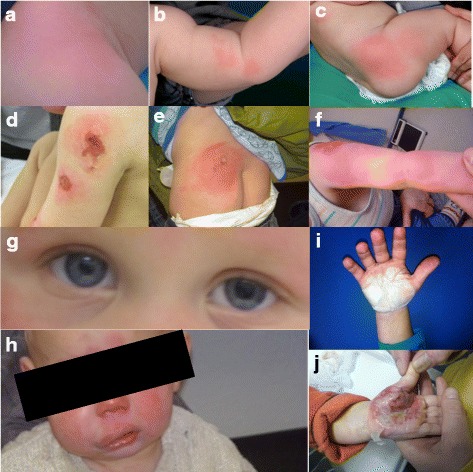
Evolution of symptoms according to age of life (in months). 5 months: Initial symptoms were erythematous skin lesions distributed over the whole body (**a**–**c**). 8 months: Macrohaematuria, epistaxis and bloody diarrhoea were reported. 9 months: Fever episodes every second week. 12 months: Deep skin lesions with excoriation and ulcerations (**d**). Anisocoria (**g**) and unilateral facial swelling with sudden occurrence and transient (**h**). 16 months: deep ulcerating or necrotizing skin lesions with impaired heeling at bone marrow puncture site (**e**) or vaccination sites (**f**). 18 months: bullous skin lesions occurred with sudden detachment of epidermal layers with predominantly palmar and plantar localisation (**i**). Blisters healed without scarring usually after 2 weeks (**j**)

We report the case of a 3 year old Caucasian boy who first developed erythematous patches distributed over the whole body at the age of 5 months (Fig. 1). A skin biopsy was performed, showing faint polymorphonuclear granulocytic infiltrates; a histopathologic diagnosis of probable autoinflammatory skin disease was made, which prompted further diagnostic evaluations into SAIDs (s. Table 1). At 8 months of age, macroscopic haematuria, bloody diarrhoea and epistaxis were reported by the mother. Shortly after, recurrent episodes of fever up to 39.5 °C, 1–3 days duration, were reported to be occurring every 2 weeks, accompanied by an increase in erythematous skin lesions. From the age of 12 months, ulcerative skin lesions developing on previously intact skin primarily on the extremities, a sudden-onset unilateral facial swelling and transient anisocoria were seen (Fig. 1). Deep skin defects developed after medical interventions such as bone marrow aspiration or immunizations, which were interpreted as representing a pathergy phenomenon. Subsequently, from the age of 18 months, palmoplantar bullous skin lesions occurred with rapid detachment of epidermal layers. One skin lesion on the foot healed with scar formation.

**Table 1:** Diagnostic testing with normal results

Autoinflammation/Autoimmunity
• Acute phase reaction proteins (CRP, SAA), Erythrocyte-Sedimentation Rate (ESR), phagocyte-specific S100-proteins
• Sanger sequencing of the following genes: NLRP3 (CAPS), TNFRSF1a (TRAPS), MVK (HIDS), MEFV (FMF), PSTPIP1 (PAPA)
• Antinuclear-antibodies (ANA), anti-dsDNA, antineutrophil cytoplasmic antibodies (ANCA)
Immunodeficiency
• Complement system tests (AP 50, CH 50, C3 and C4)
• IgG, IgM, IgA, IgG-subclasses, lymphocyte subpopulations, vaccination titers, T-cell proliferation, in vitro granulocyte respiratory burst, in vitro granulocyte phagocytosis, maternal chimerism
• Bone marrow aspiration
Dermatologic conditions
• Skin biopsies on six occasions showing mild neutrophil infiltration
• Testing for epidermolysis bullosa, epidermolysis bullosa aquisita, pemphigoid
Intoxications
• Intensive interview with family for identification of accidental contaminants (plants, detergents, dyes, insecticides, mercury exposure etc.) in their household and evaluation by public health department.
• Target-oriented tracing for contaminants in the private house of the family by the local health authority
• Investigation of patient material for contaminants (e.g. urine for heavy metals)
Miscellaneous
• Ultrasonography of abdomen and lymph nodes, cranial MRI on two occasions, echocardiography
• Diagnostic hospitalisation for surveillance of suspicious activity of the family (10 months of age; 32 months of age)
• Extensive ophthalmologic investigations for eye inflammation and reasons for anisocoria

*Abbreviations: CAPS* cryopyrin-associated periodic syndrome, *CRP* C-reactive protein, *FMF* familial mediterranean fever, *HIDS* hyper-IgD syndrome, *PAPA* pyogenic arthritis, pyoderma gangrenosum, acne, *SAA* serum amyloid A, *TRAPS* tumor necrosis factor receptor-associated periodic syndrome

The boy was the youngest of several siblings of a family where both parents were part of the household and did not have connections to the health care sector, and who had no apparent psychiatric abnormalities.

A diagnosis of SAID was one of the first diagnoses suspected, and was first proposed by the dermatologists, as fever and skin symptoms are typical findings for these entities. However, extensive testing yielded negative results (Table 1). Autoimmune diseases and primary immunodeficiencies were ruled out by these investigations. Primary dermatologic disorders such as congenital or acquired epidermolysis bullosa, bullous pemphigoid diseases and other blistering skin diseases were ruled out by skin biopsy. In total, six skin biopsies were performed over a time span of 2 years. All biopsies showed faint perivascular lymphocytic infiltrates associated with rare infiltrates of polymorphonuclear granulocytes, possibly caused by mechanical or medical irritation of the skin. Several imaging studies were performed including abdominal ultrasound, brain MRI and echocardiography, all with normal results. Eye exams did not show any inflammatory changes and an underlying cause for the episodic mydriasis was not seen. Metabolic diseases were also ruled out by extensive testing. Experts for childhood metabolic diseases speculated on the presence of a leukotriene-associated disorder, but there was no specific evidence to support this suggestion.

Due to suspecting a monogenic SAID like TRAPS or an undifferentiated SAID throughout the disease course, several treatments were implemented sequentially or in combination, including prednisolone up to 2 mg/kg/d, azathioprine (4 months of therapy), dapsone (3 months of therapy), anakinra, ibuprofen and/or hydroxychloroquine. However, most of the signs and symptoms described above persisted or recurred during treatment.

Subsequently, clinical investigations shifted increasingly to the search for external and/or toxic factors. Contaminants in the household were considered and actively investigated, and screening for heavy metal poisoning was pursued. In total five hospital admissions for diagnostic purposes took place.

As the extensive work-up performed continued to yield only negative results, it became increasingly apparent that intrinsic disease was unlikely and MBPS was suspected. However, during a 3-week hospital stay with intensified surveillance, no maternal interference, that might explain the patient’s signs and symptoms was observed, symptoms remained the same. During this time two findings in particular aroused suspicion, (1) reversible unilateral (otherwise unexplainable) mydriasis, not reactive to topical application of pilocarpine 1% and (2) the sudden appearance of bullous skin lesions in previously intact skin. A North American expert panel on child abuse was consulted by telephone conference and it was felt that this case was highly suspicious for MBPS. Ultimately, both parents were confronted with the probable diagnosis with participation of the youth welfare service and an attending psychiatrist. As the mother was the person most consistently involved in the care of the child and was always present during hospital stays, we suspected her rather than the father to be the perpetrator. The mother and patient were separated immediately and the patient was admitted together with his father. Within 2 weeks all signs and symptoms of disease resolved.

In the meantime, the parents had contacted the police to report, that a crime had been committed by an unknown perpetrator. After 6 weeks of hospitalization, the patient’s mother confessed in writing to having performed multiple actions that resulted in different signs and symptoms. These actions ranged from (1) erroneous reporting (fever; medication not given), to (2) interference of specimens (blood in urine/diaper; manipulation of thermometer), and (3) induction of symptoms (mechanical irritation; application of capsaicin, anti-wart patches, mydriatic eye drops and saliva/stool application to induce skin infection) (Table 2).

**Table 2:** Symptoms and probable causative action of the mother

Symptom	Probable etiology
erythematous skin lesions	rubbing and mechanical irritation; application of capsaicin containing ointments
macrohaematuria, bloody diarrhoea and epistaxis	addition of maternal blood
fever episodes, documented also during hospitalisation	manipulation of thermometer; deceptive reporting by the mother
ulcerations and excoriations	extensive rubbing and mechanical irritation
ulcerations after vaccinations and bone marrow puncture	mechanical manipulation
palmar and plantar bullous skin lesions	administration of anti-warts patches
angioedema in face and dorsum of hands and feet	mechanical manipulation; presumably application of capsaicin containing ointments
anisocoria	administration of mydriatic eye drops
Infections of the acoustic meatus	administration of salivate or diaper content

Retrospectively, the primary care pediatrician reported abnormally frequent visits of the mother with her son to his medical practice, with up to 12 visits a month, possibly indicating secondary gain for the mother. However, no medical opinions beyond our center were accessed by the family. At the age of 3 years, a diagnosis of MBPS was firmly established with the evidence that separating mother and child led to spontaneous resolution of all disease symptoms and signs, along with the confession from the mother.

## Review of the literature

MBPS is a potentially life-threatening disorder with up to 6% mortality. The clinical spectrum ranges from a caregiver displaying too much concern regarding minor symptoms, to a caregiver inducing illness with possible major impact on the child’s physical and mental health [8]. Factitious disorders in adolescents and adults have been described to simulate rheumatic conditions such as panniculitis or lupus [9, 10]. Such observations have been made in patients treated for lupus-like disease who were for example positive for ANA (an unspecific finding) and also had the appearance of renal symptoms (due to manipulated urine), rash (which was fabricated) along with reported photosensitivity or arthritis [10]. Nonetheless, a search of PubMed with the terms “Munchausen Syndrome by Proxy” and “Rheumatology”, “Autoinflammation”, “Autoimmunity”, “Arthritis” or “Lupus” retrieved no results for original data.

In contrast, fever was reported as early as 1987 as belonging to one of the most frequent symptoms of MBPS detected in 10% of 117 cases along with bleeding (44%), seizures (42%), CNS depression (19%), apnoea (15%), diarrhoea (11%) and vomiting (10%) [11]. Fever in MBPS can result from the induced infections, but is also falsely reported or fabricated via the manipulation of thermometers, physically generated hyperthermia, or falsified on charts [11]. Fever in MBPS has also been chemically induced by the deliberate administration of diazoxide, alimemazine, ipecac, or mercury poisoning [12–15].

MBPS mimicking rheumatic conditions is probably uncommon but may be underreported. A case-report from 1987 describes a 7-year old girl, who presented with fever of unknown origin and then repeatedly presented with remitting fever over a period of 6 years and was transiently treated as SJIA. Additional observations were asthma, chronic purulent otitis media, and unilateral mydriasis. Finally, severe diarrhoea and vomiting found to be due to theophylline intoxication, were the last symptoms before discovery of the underlying condition [16].

We are aware of another MBPS case, which was first erroneously treated for hemophagocytic lymphohistiocytosis (HLH), another rare immunologic condition associated with recurrent fevers [17]. However, the reverse has also been reported, with a case of an SAID, later identified as MVK deficiency, being mistaken as MBPS [18].

## Discussion and conclusions

The mean time from the onset of symptoms to the time of diagnosis in MBPS is around 15 months [11]. Recognition of MBPS early in the disease course is difficult and, as in this case, can be delayed for several reasons. Importantly, clinical and skin biopsy findings were misinterpreted. Therefore in this case, the popular phrase coined by Maslow “if all you have is a hammer, everything looks like a nail” applies. We were, in retrospect, focussing for too long on identifying a unifying inflammatory condition for this patient’s disease process. Even though the maternal actions (Table 2) have been described in the past in the context of MBPS, especially with fever as a key finding, the multitude of actions in this case was distracting [11, 19–21].

The importance of a multidisciplinary approach must be emphasized. In our case, the diagnosis became obvious during case conferences involving general paediatric, dermatology, immunology, rheumatology, ophthalmology, pharmacology/toxicology, psychiatrics, and forensic medicine specialists. Specific observations that led to the diagnosis included the absence of measurable inflammation even during episodes of fever, unilateral recurrent (otherwise unexplainable) mydriasis and the sudden occurrence of blisters on previously intact skin. These findings were felt to be unexplainable by any mechanism other than by MBPS.

While fever is a frequent sign associated with MBPS, there are no previous reports or case series on patients with MBPS who had been mistaken as SAIDs. On the other hand, since SAIDs comprise a heterogeneous group of inflammatory disorders that were collectively defined only around 15 years ago, an under diagnosis of SAIDs is also likely [1]. It remains a clinical dilemma that fever may be a sign of serious disease processes including SAIDs, but, as discussed above, can also be produced factitiously by various means. Therefore, critical evaluation and scrutiny of recurrent fevers in children is mandatory. A high index of suspicion both for SAID and MBPS is needed to unravel cases with atypical combinations of symptoms and a poor response to anti-inflammatory therapies.

In conclusion, we describe for the first time a clinical case of MBPS specifically mimicking SAIDs, which resulted in a delayed diagnosis. We recommend MBPS be added to the differential diagnosis of SAIDs. Inconsistent clinical findings, for example lack of measurable inflammation during fever, should prompt suspicion for diagnosis of MBPS.

## Abbreviations

CAPS: Cryopyrin associated periodic syndromes
CRP: C-reactive protein
ESR: Erythrocyte sedimentation rate
FMF: Familial Mediterranean fever
MBPS: Muenchhausen by proxy syndrome
MVK: Mevalonate kinase deficiency
SAA: Serum amyloid A
SAIDs: Systemic autoinflammatory diseases
TRAPS: Tumor necrosis factor receptor associated periodic syndrome
